# Diagnostic and therapeutic odyssey of two patients with compound heterozygous leptin receptor deficiency

**DOI:** 10.1186/s40348-020-00107-3

**Published:** 2020-11-03

**Authors:** Stefanie Zorn, Julia von Schnurbein, Katja Kohlsdorf, Christian Denzer, Martin Wabitsch

**Affiliations:** Center for Rare Endocrine Diseases, Division of Paediatric Endocrinology and Diabetes, Department of Paediatrics and Adolescent Medicine, Ulm University Medical Centre, 89075 Ulm, Germany

**Keywords:** Leptin receptor, Genetics, Monogenetic obesity, Early-onset obesity, Hyperphagia

## Abstract

**Background:**

Rare genetic variations in the leptin-melanocortin signalling pathway can severely impair appetite regulation and cause extreme obesity in early childhood.

**Case presentation:**

Our case reports describe the diagnostic and therapeutic procedures in a girl as well as in a non-related boy of non-consanguineous, German parents with severe early-onset obesity, pronounced hyperphagia, and permanent food-seeking behaviour. Excessive weight gain within the first year of life initiated extensive diagnostics without finding a causal diagnosis. Furthermore, a wide range of intensive, interdisciplinary, and behavioural therapies for weight control were unsuccessful. Prior to bariatric surgery, the 18-year-old girl and the 14-year-old boy reached a BMI of 67.7 kg/m^2^ and 55.2 kg/m^2^, respectively. However, even surgical outcomes were unsatisfactory. A subsequently initiated genetic analysis including sequencing of the leptin receptor gene revealed compound heterozygous variants as a cause of the severe early-onset obesity in both patients (c.2598-3_2607delTAGAATGAAAAAG and c.2227 T>C; c.1874G>A and c.2051A>C). Both patients were enrolled in the clinical study RM-493-015 and treated with melanocortin receptor agonist setmelanotide. Currently, they are still on setmelanotide treatment in the extension trial RM-493-022.

**Conclusion:**

Our case report illustrates the urgent necessity of early genetic diagnostics in children with severe early-onset obesity to avoid frustrating and potentially damaging therapies. Thus, genetic examination should precede bariatric surgery. In the future, several pharmacological therapies will be available for some forms of monogenetic obesity.

## Background

Obesity in children and adolescents is associated with an increased risk for serious health consequences and with an impaired psychological and social development [1, 2]. Severe, early-onset obesity can be due to disease-causing genetic variations. These can interrupt the leptin-melanocortin signalling pathway and impair the regulation of hunger and satiety [3–5]. There is growing knowledge about the effects of rare genetic variations on body weight and new pharmacological treatments for children with monogenic obesity, e.g. leptin receptor deficiency will become available. It is therefore important to identify monogenic obesity early in life in order to prevent subsequent secondary damages [6–8].

## Case presentations

### Patient 1

**Fig. 1:**
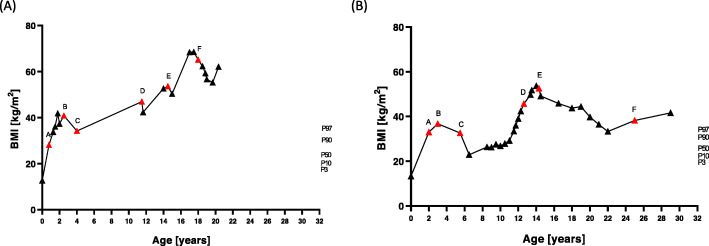
Body mass index (BMI) trajectories from birth to diagnosis of leptin receptor deficiency in patient 1 (**a**) and patient 2 (**b**). Previous therapy approaches to treat obesity are marked in red (A–F) and can be seen in Table 2

The girl is the second child of German, non-consanguineous parents. She was a newborn of 39 weeks of gestational age, weighted 3690 g, and had a birth length of 54 cm. Already in the first weeks of her life, the girl showed signs of hyperphagia by crying regularly after the end of a meal and only calmed down after further food intake. A perceptible feeling of satiety did not occur even after extensive food intake. Excessive weight gain within the first year of life led to a body weight of 18 kg (BMI 28.1 kg/m^2^, BMI SDS 6.2) (Fig. 1a). Despite numerous diagnostic examinations, no causal diagnosis could be found for the extreme early-onset obesity (Table 1). The persistent desire for food and the continuous weight gain (Fig. 1a) played a major role in daily family life and massively distressed the girl and her family. Negative social experiences at school (e.g. bullying and teasing) led to social isolation. Intensive, interdisciplinary, behavioural treatments to reduce weight were unsuccessful in the long term (Table 2). Repeated setbacks after frustrating weight loss attempts caused a deep depression with suicidal thoughts, but no drug treatment was required. After exhaustion of common therapeutic approaches, she finally decided to undergo bariatric surgery at the age of 18 years. At this time, she weighed 188 kg (BMI 67.7 kg/m^2^). As a result, she lost 38 kg within 1 year followed by rapid weight regain of 22 kg within 8 months (Fig. 1). However, hypogonadotropic hypogonadism diagnosed at the age of 17 resolved one year after bariatric surgery.

**Table 1:** Chronology of diagnostic examinations performed in the two patients to exclude endocrine and syndromic diseases causing severe, early-onset obesity

	Age, year (y) month (m)	Suspected diagnoses	Methods
**Patient 1**	0 y 9 m	Hypercortisolism	Cortisol profile and dexamethasone suppression test
		Hypothyroidism	Thyrotropin-releasing hormone stimulation test
		Growth hormone deficiency	Insulin hypoglycaemia test and radiography
		Brain tumour/brain malformation	Sonography
	1 y 10 m	Prader-Willi syndrome	MS-MLPA
		Chromosome aberrations	Chromosome analysis
		Congenital leptin deficiency	Leptin ELISA
	2 y 6 m	Brain tumour/brain malformation	MRI
		Pituitary tumour	MRI
		Hypercortisolism	Urinary cortisol profile
		Disorder of the adipocyte differentiation	In vitro functional examination
		Adrenal insufficiency	Adrenocorticotropic hormone test
		Adrenal gland tumour	CT
		Congenital leptin deficiency	Leptin ELISA
		Prohormone convertase deficiency	Oral glucose tolerance test, proinsulin ELISA
**Patient 2**	1 y 11 m	Hypercortisolism	Cortisol profile
		Hypothyroidism	Laboratory
		Chromosome aberrations	Chromosome analysis
		Insulinoma	Blood glucose profile
		Prader-Willi syndrome	MS-MLPA
		Pseudohypoparathyroidism	Laboratory values and radiography
		Brain tumour/brain malformation	CT
		Glycogenosis type I	Clinical examinations
	2 y 9 m	Brain tumour/brain malformation	MRI
		Hypothalamus tumour	MRI
		Prader-Willi syndrome	MS-MLPA
	5 y 6 m	Eating disorders	Psychological examinations
	8 y 11 m	Growth hormone deficiency	l-Arginine and insulin-tolerance tests
	9 y 3 m	Anterior pituitary insufficiency	Corticotropin-releasing hormone stimulation test and thyrotropin-releasing hormone stimulation test
		Brain tumour/brain malformation	MRI
	11 y 9 m	Pituitary tumour	MRI
	12 y 2 m	Disorder of the hypothalamic-pituitary-adrenal axis	Combined pituitary stimulation test
		Adrenal insufficiency	Adrenocorticotropin stimulation test

*CT* computer tomography, *ELISA* enzyme-linked immunosorbent assay, *MS-MLPA* methylation-specific multiplex ligation-dependent probe amplification, *MRI* magnetic resonance imaging

**Table 2:** Measures for treatment of obesity from birth to the age at diagnosis of leptin receptor deficiency in the two patients

	Age, year (y) month (m)	Measures for treatment of obesity	Impact on weight
**Patient 1**	Ongoing	Restrictive food intake and motivation for physical activity	None
	0 y 9 m	9 days stay in a paediatric clinic with caloric restriction to 600 kcal per day^a^	None
	3 y	21 days stay in a specialised clinic for metabolic disorders and Prader-Willi syndrome^b^	None
	4 y 2 m	14 days stay in a specialised clinic for Tomatis Listening Therapy^c^	None
	10 y 4 m	2 years outpatient psychotherapy for mother and child	None
	11 y 5 m	6 weeks stay in a rehabilitation clinic focusing obesity^d^	− 10.7 kg (− 9.86%), followed by weight regain
	14 y 7 m	6 months stay in a rehabilitation clinic with obesity long-term therapy^e^	− 15.6 kg (− 6.41%), followed by 9.3 kg weight regain within 1 month
	18 y 1 m	Leaving the parental home and moving into an assisted living community	− 8 kg (+ 4.26%)
	18 y 3 m	2 years outpatient psychotherapy	None
	18 y 4 m	Bariatric surgery: sleeve gastrectomy^f^	− 38 kg (− 20.13%), followed by weight regain (overall weight reduction, 16 kg (− 8.47%))
**Patient 2**	Ongoing	Restrictive food intake and motivation for physical activity	None
	1 y 11 m	15 days stay in a paediatric clinic with daily 800 kcal caloric restriction^a^	None
	2 y 9 m	7 weeks stay in a paediatric clinic with daily 400 kcal caloric restriction^b^	− 4 kg (− 12.05%)
	3 y 10 m	1.5 years outpatient psychotherapy	None
	5 y 6 m	1 year inpatient psychotherapy^c^	− 14 kg (− 29.79%), followed by slow weight regain
	6 y 5 m	3 years outpatient psychotherapy	None
	9 y 5 m	3 years growth hormone treatment due to short stature and growth hormone deficiency	+ 59 kg (+ 38.8%)
	12 y 7 m	4 months stay in a rehabilitation clinic with obesity long-term therapy^d^	+ 4 kg (+ 4.0%)
	12 y 11 m	Regular advice and support from the youth welfare department, psychological care and medical weight control	None
	14 y 3 m	Bariatric surgery: gastric banding^e^	− 49 kg (− 33.84%)
	14 y 4 m	28 days stay in a child and adolescent psychiatry after gastric banding	− 5 kg (− 3.72%)
	25 y 0 m	Removal of gastric banding due to band migration^f^	+ 15 kg (+ 14.8%) within 4 years

^a–f^Refer to Fig. 1 and display the time points of interventions and their impact on weight

### Patient 2

The boy was born with a gestational age of 40 weeks, a birth weight of 3320 g, and a birth length of 50 cm. He is the second child from healthy, non-consanguineous parents of German-Polish origin. Since the first year of life, the boy had permanent hunger, followed by excessive weight gain. Consequently, he weighed 25 kg at the age of 2 years (BMI 33.0 kg/m^2^, BMI SDS 5.5). His extreme body weight restricted his mobility and delayed age-appropriate gross motor development (Fig. 1b). Therefore, comprehensive diagnostics were performed to find the cause of the early-onset obesity. However, no causal diagnosis could be found (Table 1). At the age of 8 years, short stature and growth hormone deficiency were diagnosed. During 3 years of growth hormone treatment, he caught up to a height within normal range from 1.24 to 1.55 m but he also gained weight from 42.5 to 101.5 kg. He attempted a number of conservative treatment approaches to reduce weight without any appreciable success (Table 2). He behaved dissocially and aggressively towards peers and adults, partly in response to negative social experiences, accusations, and teasing. The familiar atmosphere was extremely tense due to his permanent demand for food, his extreme obesity, and the lack of a medical explanation. Finally, he developed a deep depression with suicidal thoughts as a result of the immense physical, mental, familiar, and social stresses. However, he required no drugs to treat depression. At the age of 14 years and a body weight of 154 kg (BMI 55.2 kg/m^2^), he received gastric banding which helped him to lose 16 kg within one year. In the same year as bariatric surgery, he obtained the diagnosis of hypogonadotropic hypogonadism, which could no longer be detected 4 years after gastric banding. In total, he lost 49 kg within 8 years after gastric banding. This weight reduction seems to be impressive, but it should be noted that the follow-up process after gastric banding was associated with severe complications. Hence, he suffered from recurrent episodes of severe epigastric pains, nausea, vomiting, and diarrhoea. This could also have contributed to the impressive weight reduction. In total, the complications of the gastric banding lead to a deterioration in quality of life. At the age of 25 years, the gastric band had to be removed due to gastric band migration with severe epigastric pains, nausea, and vomiting (Table 2).

### Findings

At the time of diagnosis, patient 1 is 20 years old, lives on her own, and is completing a training. She weighs 172 kg and has numerous obesity-associated comorbidities (dyslipidaemia, hyperinsulinaemia, hepatic steatosis, hyperuricaemia, and a sleep apnea syndrome). Furthermore, she has a hypochromic microcytic anaemia due to iron deficiency and vitamin D deficiency.

Patient 2 is 29 years old, is unemployed, and lives with his girlfriend in a shared apartment. He is holding a disabled person’s pass with a degree of disability of 60%. He weighs 120.5 kg 4 years after gastric band removal. Apart from obesity, he suffers from bronchial asthma, atopic dermatitis, and episodes of recurrent diarrhoea and vomiting.

Selected clinical and laboratory findings in the two patients are presented in Table 3.

**Table 3:** Clinical and laboratory findings at the time of diagnosis

		Patient 1	Patient 2	Reference range
Age		20 years	29 years	
**Weight**	kg	172	120.5	
**Height**	m	1.66	1.70	
**BMI**	kg/m^2^	62.1	41.6	
**BMI SDS**		3.64	3.07	
**Blood pressure**	mmHG	121/85	137/86	
**Pubertal stage by Tanner**		PH, 5; B, 5	PH, 6; testicular volume, 16 ml and 14 ml	
**Insulin-like growth factor 1**	ng/ml	231	182	f, 122–384; m, 117–321
**Leptin**	μg/l	34.4	12.5	3.6–11.1
**Total cholesterol**	mmol/l	4.3	3.8	< 5.0
**LDL cholesterol**	mmol/l	3.2	2.3	> 1.0
**HDL cholesterol**	mmol/l	0.5	1.2	< 3.0
**Thyreotropin**	MIU/l	2.02	1.88	12.8–20.4
**Free thyroxine**	pmol/l	11.7	19	3.13–6.76
**Testosterone**	μg/l	0.19	4.25	f, 0.084–0.481; m, 2.490–8.360

*BMI* body mass index, *BMI SDS* body mass index standard deviation score, *f* female, *LDL* low-density lipoprotein, *HDL* high-density lipoprotein, *m* male

### Diagnosis

Numerous endocrinological and syndromic diseases known to cause early-onset obesity were excluded in both patients (Tables 1 and 2). Moreover, it seemed inappropriate to attribute the extreme early-onset obesity solely to an unfavourable lifestyle. Based on recent findings on monogenetic causes of obesity, molecular genetic examinations were initiated. For molecular genetic diagnostic, the genomic DNA of both patients was isolated from leucocytes. Subsequently, the DNA was amplified by polymerase chain reaction for all coding exons from the leptin receptor (LEPR) gene and then sequenced by Sanger method. The available sequence data were compared with the common LEPR reference sequence (NM_002303.4). The results show that compound heterozygous variants in the *LEPR* gene are the cause for the severe early-onset obesity in both patients. Patient 1 has a splice site/frameshift deletion (c.2598-3_2607delTAGAATGAAAAAG) as well as a missense mutation (c.2227 T>C [p.Ser743Pro]). Patient 2 carries the missense mutations c.1874G>A [p.His684Pro] and c.2051A>C [p.Trp625*]. These changes in the *LEPR* gene result in both patients in a premature termination of the protein biosynthesis and a loss of LEPR function.

Disruptions in the leptin-melanocortin signalling pathway have far-reaching consequences for the regulation of hunger, satiety, and body weight [4, 9]. The hormone leptin is secreted by the adipose tissue and reflects the energy stores of the body. Leptin crosses the blood-brain barrier and activates LEPR in the hypothalamus, initiating a complex signalling cascade. Consequently, leptin inhibits the production of the neuropeptide Y and agouti-related peptide in orexigenic neurons, while stimulating the proopiomelanocortin (POMC) synthesis in anorexigenic POMC neurons. POMC is then processed via prohormone convertase 1 into the α-melanocyte-stimulating hormone (α-MSH), which activates the melanocortin 4 receptor (MC4R). This finally mediates a feeling of satiety, reduces food intake, and enhances energy expenditure [10–12]. In summary, the disease-causing variants in the *LEPR* gene found in the two patients interrupt the leptin-melanocortin signalling pathway and lead to severe early-onset obesity, pronounced hyperphagia, and permanent food-seeking behaviour.

### Treatment

The finding of the causative molecular diagnosis meant a great relief to the patients and their families. However, so far, there is no standardised causal treatment option for patients with disease-causing variants in the *LEPR* gene. Both patients could not achieve weight control by a balanced, calorie-reduced diet with increased physical activity. Recently, a first causal pharmacological treatment has been described [8] and is currently being evaluated in the phase III trial RM-493-015 and the extension trial RM-493-022. Both patients were enrolled in these clinical studies and started the treatment with the new MC4R agonist setmelanotide. Setmelanotide affects the leptin-melanocortin signalling pathway and thus the regulation of hunger and satiety by substituting the missing endogenous α-MSH. It activates the MC4R and induces a feeling of satiety, reduces food intake, and finally leads to a significant weight reduction in patients with LEPR deficiency [8].

## Discussion

The two case reports describe the frustrating diagnostic and therapeutic history of two patients with disease-causing, compound heterozygous variants in the *LEPR* gene. Comparable with the previous published 88 patients with LEPR deficiency, our patients show extreme early-onset obesity, pronounced hyperphagia, and hypogonadotropic hypogonadism [9, 13]. Moreover, we observed a severe psychological strain in our patients, including stigmatisation, social isolation, and severe accusations. Patients with monogenic obesity and their families require specialised care as well as comprehensive and lasting support to deal with the numerous challenges and consequences of this disease [2, 14].

It is therefore highly recommended to perform early, extensive diagnostic, including a molecular genetic testing in children with extreme obesity to provide them with the necessary support and therapy as early as possible [5, 12]. Genetic examinations for variants in the leptin gene and *LEPR* gene are particularly indicated for children at the age of 2 years with BMI ≥ 27 kg/m^2^ or at the age of 5 years with BMI ≥ 33 kg/m^2^. In addition, in children with BMI > 99.5 age- and sex-adjusted percentile, and normal weight parents or hyperphagic eating behaviour, a genetic investigation for other monogenic variations causing obesity should be carried out [15].

The main objective in the treatment of patients with monogenic obesity is to control body weight. This should primarily be achieved through a balanced, energy-reduced, controlled diet while increasing physical activity. Our case reports show however that behavioural-based treatment programmes in specialised obesity clinics neither in an outpatient nor in an inpatient setting contributed to weight control. On the contrary, they lead to frustration and disappointment. Bariatric surgery also did not result in long-term success of weight loss and weight maintenance in our patients comparable to three other published case reports of patients with LEPR deficiency and bariatric surgery [16, 17]. To date, only one patient with LEPR deficiency was able to reduce his weight in the long term due to bariatric surgery [18]. Currently, the evidence for the efficacy of bariatric surgery is limited in patients with LEPR deficiency and other monogenic obesity disorders. Therefore, bariatric surgeries should only be conducted in patients with monogenic obesity after a critical risk-benefit analysis and after the exhaustion of the existing non-invasive, conservative, and pharmacological treatment options [19, 20]. In order to avoid unsatisfactory bariatric therapies, genetic testing should be performed in patients with hints for monogenic obesity to exclude monogenic causes for obesity preoperatively. This especially includes severely obese patients whose weight development in early childhood is striking and whose BMI exceeded 27 kg/m^2^ at the age of 2 years and 33 kg/m^2^ at the age of 5 years [15]. The onset of obesity at school age is less frequently associated with monogenic obesity [15].

The surprising disappearance of hypogonadotropic hypogonadism could probably be attributed to the postoperative initial weight loss after bariatric surgery in our patients, as well as in two other patients with LEPR deficiency [16, 18]. However, there are also reports of spontaneous puberty in patients with LEPR deficiency without surgery. Furthermore, a gender-specific effect on reproduction is currently discussed in patients with LEPR deficiency [21].

So far, only individual pharmacological treatment approaches such as off-label use of methylphenidate provided the possibility to support weight control in addition to conventional therapies containing strict nutritional and exercise control [7]. However, a first causal treatment option with the MC4R agonist setmelanotide is recently available in the context of clinical studies for certain patients with monogenic obesity. The first results of patients with LEPR deficiency under setmelanotide treatment showed significant reductions in body weight and hyperphagia with only mild adverse events [8]. Thus, setmelanotide currently offers a promising causal treatment option for certain patients with monogenic obesity.

## Lessons learned and specific recommendations

Case reports on rare genetic diseases as demonstrated in our paper are important to improve knowledge and to derive specific recommendations for the clinic. Therefore, we summarise the lessons learned from the two patients with LEPR deficiency and provide some recommendations for diagnosis and treatment of patients with suspected monogenic obesity, especially for patients with LEPR deficiency.

Severely obese patients should receive genetic diagnostic as early as possible if they show suspicious weight development in early childhood [15]. If no genetic cause for the extreme obesity can be found in a molecular genetic obesity panel analysing all currently established genes causative for monogenic obesity, further genetic diagnostics should be performed. It is possible to find new or further causes for genetic obesity using microarray-based comparative genomic hybridization, methylation-specific multiplex ligation-dependent probe amplification, or whole exome sequencing of the patient and parents (trio analysis).

After diagnosis of monogenic obesity, e.g. LEPR deficiency, further diagnostic examinations to find the cause of obesity are dispensable. If a genetic cause of obesity has been diagnosed, further examinations related to comorbidities should be carried out according to the recent guidelines [22]. The screening for comorbidities in paediatric obesity should include the screening for diabetes mellitus, dyslipidaemia, hypertension, non-alcoholic fatty liver disease, obstructive sleep apnea, etc. [22].

There are causal pharmacological treatment options for some forms of monogenic obesity, e.g. patients with leptin deficiency can be successfully treated with metreleptin [6]. In the future, one or more drugs targeting the leptin-melanocortin signalling pathway will be available to treat patients with LEPR deficiency and POMC deficiency [8, 23].

A specific and individually tailored follow-up and support plan is recommended for patients with genetic obesity without causal therapy or extremely obese patients with negative finding of genetic obesity after extensive molecular genetic diagnostics. All specialists involved in the treatment should be aware that patients with diagnosed monogenic obesity are exposed to a strong biological basis that commonly causes the onset of obesity in early childhood. Therefore, it should be avoided to blame the patient or family for the extreme weight gain. Furthermore, psychological support should be a priority in the individual follow-up and support plan of these patients. Psychologist should primarily help and support patients and parents to deal with stigmatisation and discrimination in the social environment and in the public health sector. In addition, it is essential to provide a healthy and restricted diet as well as daily physical activity. Training programmes focusing on positive feedback can be helpful in achieving daily physical activity and increasing self-empowerment, self-confidence, and general well-being. Specialists involved in treatment should ensure that the support programmes offered to the family are individually tailored to the requirements of the patient and family. Unrealistic treatment goals must be avoided in patients with monogenic obesity particularly with regard to weight reduction. Patients with monogenic obesity and currently no causal pharmacological treatment option may be treated with an off-label use of methylphenidate and dextroamphetamine to support weight control and prevent further weight gain [7, 24]. Additionally, the involvement of a social worker can support the patient and family at home to deal with the daily challenges associated with this disease and the extreme obesity. Those specialists can provide diverse socio-medical assistance based on the new International Classification of Functioning, Disability and Health (ICF) core set for obesity. The ICF core set defines the spectrum of symptoms and problems in functioning of persons with obesity. On this basis, obese patients in some countries can receive government support to encourage social participation of extremely obese children and their parents [2].

## Conclusion

These two case reports illustrate the urgent necessity for early molecular genetic diagnostics in patients with severe, early-onset obesity to avoid lengthy diagnostic and unsuccessful and frustrating therapeutic procedures. A BMI > 27 kg/m^2^ at the age of 2 years indicates causative biallelic variants in the genes encoding leptin or leptin receptor. In addition, a BMI > 99.5 age- and gender-adjusted percentile and pronounced hyperphagia indicate a monogenic disease. Particularly, in these patients, genetic testing is highly recommended. Furthermore, it is advisable to perform genetic testing before bariatric procedures in young patients with early-onset, severe obesity who might carry a causative genetic variant to avoid unsatisfactory results in such patients. After the diagnosis of monogenetic obesity, it is very important that patients are seen by specialists to monitor weight and comorbidities and to obtain the necessary therapeutic and psychological support. In the future, causal pharmacological therapies will be available for patients with different forms of monogenic obesity.

## Data Availability

The datasets generated and analysed during the current case report are available from the corresponding author on reasonable request.

## Abbreviations

α-MSH: α-Melanocyte-stimulating hormone
BMI: Body mass index
ICF: International Classification of Functioning, Disability and Health
LEPR: Leptin receptor
MC4R: Melanocortin 4 receptor
POMC: Proopiomelanocortin
